# Lung ultrasound and procalcitonin, improving antibiotic management and avoiding radiation exposure in pediatric critical patients with bacterial pneumonia: a randomized clinical trial

**DOI:** 10.1186/s40001-024-01712-y

**Published:** 2024-04-06

**Authors:** Carmina Guitart, Sara Bobillo-Perez, Javier Rodríguez-Fanjul, José Luis Carrasco, Pedro Brotons, Maria Goretti López-Ramos, Francisco José Cambra, Mònica Balaguer, Iolanda Jordan

**Affiliations:** 1 Paediatric Intensive Care Unit, Hospital Sant Joan de Déu, University of Barcelona, Passeig de Sant Joan de Déu, 2, 08950 Esplugues de Llobregat, Barcelona Spain; 2 Immunological and Respiratory Disorders in the Pediatric Critical Patient Research Group, Institut de Recerca Sant Joan de Déu, University of Barcelona, Barcelona, Spain; 3 Pediatric Infectious Diseases Research Group, Institut de Recerca Sant Joan de Déu, Santa Rosa 39-57, 08950 Esplugues de Llogregat, Spain; 4 Neonatal Intensive Care Unit, Department of Pediatrics, Hospital Germans Trias i Pujol, Autonomous University of Barcelona, Badalona, Spain; 5 Department of Basic Clinical Practice, University of Barcelona, Barcelona, Spain; 6 Consorcio de Investigación Biomédica en Red de Epidemiología y Salud (CIBERESP), Madrid, Spain; 7 School of Medicine and Health Sciences, Universitat Internacional de Catalunya, Barcelona, Spain; 8 Pharmacy Department, Hospital Sant Joan de Déu, University of Barcelona, Barcelona, Spain

**Keywords:** Childhood pneumonia, Lung ultrasound, Procalcitonin, Antibiotic prescription, Radiation, Costs, Pediatric critical care

## Abstract

**Background:**

Pneumonia is a major public health problem with an impact on morbidity and mortality. Its management still represents a challenge. The aim was to determine whether a new diagnostic algorithm combining lung ultrasound (LUS) and procalcitonin (PCT) improved pneumonia management regarding antibiotic use, radiation exposure, and associated costs, in critically ill pediatric patients with suspected bacterial pneumonia (BP).

**Methods:**

Randomized, blinded, comparative effectiveness clinical trial. Children < 18y with suspected BP admitted to the PICU from September 2017 to December 2019, were included. PCT was determined at admission. Patients were randomized into the experimental group (EG) and control group (CG) if LUS or chest X-ray (CXR) were done as the first image test, respectively. Patients were classified: 1.LUS/CXR not suggestive of BP and PCT < 1 ng/mL, no antibiotics were recommended; 2.LUS/CXR suggestive of BP, regardless of the PCT value, antibiotics were recommended; 3.LUS/CXR not suggestive of BP and PCT > 1 ng/mL, antibiotics were recommended.

**Results:**

194 children were enrolled, 113 (58.2%) females, median age of 134 (IQR 39–554) days. 96 randomized into EG and 98 into CG. 1. In 75/194 patients the image test was not suggestive of BP with PCT < 1 ng/ml; 29/52 in the EG and 11/23 in the CG did not receive antibiotics. 2. In 101 patients, the image was suggestive of BP; 34/34 in the EG and 57/67 in the CG received antibiotics. Statistically significant differences between groups were observed when PCT resulted < 1 ng/ml (*p* = 0.01). 3. In 18 patients the image test was not suggestive of BP but PCT resulted > 1 ng/ml, all of them received antibiotics. A total of 0.035 mSv radiation/patient was eluded. A reduction of 77% CXR/patient was observed. LUS did not significantly increase costs.

**Conclusions:**

Combination of LUS and PCT showed no risk of mistreating BP, avoided radiation and did not increase costs. The algorithm could be a reliable tool for improving pneumonia management.

*Clinical Trial Registration*: NCT04217980.

**Supplementary Information:**

The online version contains supplementary material available at 10.1186/s40001-024-01712-y.

## Background

Pneumonia in children is a major public health and an economic problem with a considerable impact on morbidity and mortality. It is one of the most common causes of admission to pediatric intensive care units (PICU) worldwide [1]. Its etiology can be bacterial, viral, or fungal infection [2]. As defined in the British Thoracic Society (BTS) guidelines [3], bacterial pneumonia (BP) should be considered in children when there is persistent fever > 38.5 ºC together with chest recessions and raised respiratory rate, in a previously healthy child [4–6]. However, those symptoms and signs may overlap between the different etiologies [7, 8], being a challenge to identify patients with BP [9], which is crucial concerning antibiotic use. Moreover, children are more sensitive to radiation than adults. Radiation exposure can induce non-lethal cell transformation that may become malignant after a long latency period (several years to decades) [10, 11]. It is assumed that a linear relationship may exist between exposure and cancer risk, with no threshold value below which this risk is zero. Based on this, the probability of developing cancer is presumed to increase with radiation dose even for low-dose medical imaging procedures [10]. Because of this, health care providers should be careful to reduce radiation exposure to pediatric patients [12].

The presence of a new infiltrate on the chest X-ray (CXR) supports the definitive diagnosis of pneumonia [13]. However, although CXR is considered the best diagnostic option in children, it shows low specificity regarding etiology diagnosis [14, 15]. Besides, it has several limitations: it exposes patients to radiation and has limited diagnostic accuracy for community-acquired pneumonia (CAP) (54% sensitivity and 57% specificity, using chest-CT as a gold standard) [16]. On the other hand, lung ultrasound (LUS) has been used to analyze pleural and lung tissue pathological conditions. The available evidence suggests that LUS is a reliable, valuable, and alternative method to CXR for the diagnosis of pediatric pneumonia [15, 16]. It can be performed at the patient’s bedside (point-of-care) and is radiation free [17, 18].

Microbiological diagnosis should be attempted in children with severe pneumonia who require intensive care, in order to differentiate between viral and bacterial etiology and optimize antibiotic use. However, studies regarding pneumonia etiology are challenging due to the low yield of blood cultures, the inconvenience to obtain adequate sputum specimens, and the reluctance to perform lung aspiration and bronchoalveolar lavage (BAL) in children [19]. Over the last decade, polymerase chain reaction (PCR) techniques have developed considerably and improved viral and bacterial detection. However, they are not always available or accurate enough for pneumonia diagnosis etiology [20, 21].

The use of biomarkers may be helpful to elucidate the etiology of infection and identify patients with respiratory infections potentially requiring antibiotics. Procalcitonin (PCT) and C-reactive protein (CRP) are the most extensively studied biomarkers. PCT usefulness has been proven for bacterial infection diagnosis [22, 23], helping to differentiate it from viral infection [24–26]. PCT increases earlier than CRP in the presence of an active infection and decreases quickly when the infection is controlled, allowing monitor antibiotic response, potentially reducing antibiotic treatment indication and length [27–29]. However, PCT values alone may not confirm the diagnosis of BP [30]. Thus, more evidence is required to confirm the impact of PCT-guided therapy on antibiotic prescription rates among patients with respiratory infections.

Optimal antibiotic use is crucial because of the rising antibiotic resistance and the lack of new antimicrobial development. An association between antibiotic use and the appearance and spread of resistant strains has been established [31, 32]. Reducing its inappropriate use is essential [33, 34]. Therefore, it is important to search for tests or techniques that may help physicians diagnose BP.

Results of this clinical trial, regarding the accuracy of the algorithm for BP diagnosis in pediatric patients, have been previously published in the literature [35].

The present study aimed to analyze if the new diagnostic algorithm combining LUS and PCT improves the quality of BP management regarding antibiotic use, radiation exposure, and associated costs.

## Methods

### Study design and patients

PROLUSP (PROcalcitonin and Lung UltraSound algorithm to diagnose severe Pneumonia in critical pediatric patients) study, was a randomized, blinded, controlled clinical trial. It was conducted in a PICU of a tertiary pediatric hospital (Hospital Sant Joan de Déu, Barcelona, Spain) from September 2017 to December 2019 [36]. The study was conducted in accordance with the Declaration of Helsinki and approved by the local Healthcare Ethics Committee and the Institutional Review Board (code: PIC-139-16). Trials.gov registry registration number: NCT04217980. The present manuscript is a post hoc study.

Eligible patients were children under 18 years old with suspected BP. Patients with underlying pathologies such as cystic fibrosis or immunocompromised, patients previously included in the study as CAP who developed nosocomial pneumonia (NP), or patients whose CXR was evaluated by the pediatrician-researcher before the PICU admission were excluded. Refusal of parental consent, loss of protocol adherence, and death were considered withdrawal and abandonment criteria. Thus, these patients were also excluded from the study. Written parental informed consent was mandatory. Demographic information, clinical characteristics, laboratory, microbiological and radiological findings were collected [36].

### Definitions

In accordance with the British Thoracic Society guidelines [3], CAP in children is defined as the presence of compatible clinical signs and symptoms (fever, cough, tachypnea, shortness of breath) and/or abnormal respiratory auscultation (hypoventilation, tubular breath sounds, murmur), and/or thoracic or abdominal pain in a previously healthy child due to an infection acquired outside the hospital [3, 4]. BP should be considered when there is persistent fever > 38.5 ºC, with chest recession and raised respiratory rate. The appearance of an infiltrate on the X-ray and the increase in acute phase reactants levels are thought to be secondary to bacterial cause. Etiological studies through respiratory and blood samples should be attempted in those patients with suspected BP admitted to the PICU [3].

NP was suspected when pneumonia appeared after 48 h of hospitalization or within 7 days of hospital discharge. Ventilator-associated pneumonia (VAP) was suspected when pneumonia occurred in a patient receiving mechanical ventilation for more than 48 h. NP, including hospital-acquired BP and ventilator-associated BP, was characterized based on CDC’s definition [37, 38].

### LUS and CXR procedures complications

LUS was executed using a 12-MHz linear prove by a LUS trained intensive care physician who had at least 3 years of experience [39]. LUS exam was performed with the patient in supine position and, following international recommendations, 6 areas of each hemithorax were systematically scanned: superior and inferior quadrants of each anterior, lateral, and posterior zones [40]. Different patterns of aeration definitions and pneumonia diagnosis were based on previous publications [41–43]. BP pattern was considered when a lung consolidation with an air bronchogram and the presence of white lung were observed [43, 44]. Viral pneumonia (VP) pattern was based on the presence of diffuse or coalescent B lines with small, multiple, and bilateral subpleural consolidations [43]. Atelectasis were defined as those consolidations with a tissue-like pattern with static air bronchogram [43, 45].

The CXR was reviewed by an experienced consulting radiologist who specializes in pediatric imaging and pulmonary pathologies. The diagnostic criteria for BP and VP were based on previously published studies [46]. The BP pattern [47, 48] was characterized by the presence of a consolidation, which may or not present air bronchogram. Secondly, the VP pattern [49, 50] was identified by peri-bronchial infiltrates, peri-bronchial cuffing, peri-bronchial thickening or increased interstitial markings. Atelectasis [51] diagnosis was based on the presence of peripheral linear opacities, often associated with reduced lung volume.

The evaluation of radiation exposure from CXR was conducted. The assessable radiation exposure was determined by considering two factors: (1) the number of CXR that were not performed during the follow-up in comparison to the number of LUS examinations conducted during the same period, (2) the overall reduction in CXR performed in the PICU, before and after the algorithm implementation.

Complications related to image tests, including both CXR and LUS, were defined as bradycardia, desaturation, increased need for oxygen administration, increased sedation requirement, and the development of nosocomial infection as a result of the techniques used. These complications were registered during the assessment.

### PCT values and microbiological samples

The PCT values were measured using the LumiTest®PCT immune-luminometric assay from ATOM S.A. (Brahms Diagnostica GmbH). PCT levels were evaluated at the time of recruitment in both groups. A PCT value below 1 ng/mL was considered normal, while a PCT value equal to or greater than 1 ng/mL was considered indicative of a bacterial infection based on previous studies [30, 37, 52, 53].

The viral identification of the causative microorganisms was conducted using a multiplex PCR technique, which involves DNA amplification through polymerase chain reaction to detect multiple viruses. This testing was performed on nasopharyngeal aspirate (NPA) samples, or in intubated patients, on a tracheal aspirate (TA)/bronchoalveolar lavage (BAL) samples.

Cultures performance was indicated when CAP or NP were suspected. In intubated patients, the TA/BAL sample was collected once intubation was performed. To differentiate between colonization and infection, the quantification of CFU/ml was utilized. Infection was considered when there was a growth of more than 10^5^ CFU for TA and more than > 10^4^ CFU for BAL.

For blood cultures (BC), a positive result was indicated by the isolation of microorganisms from the sample.

### Randomization and interventions

**Fig. 1:**
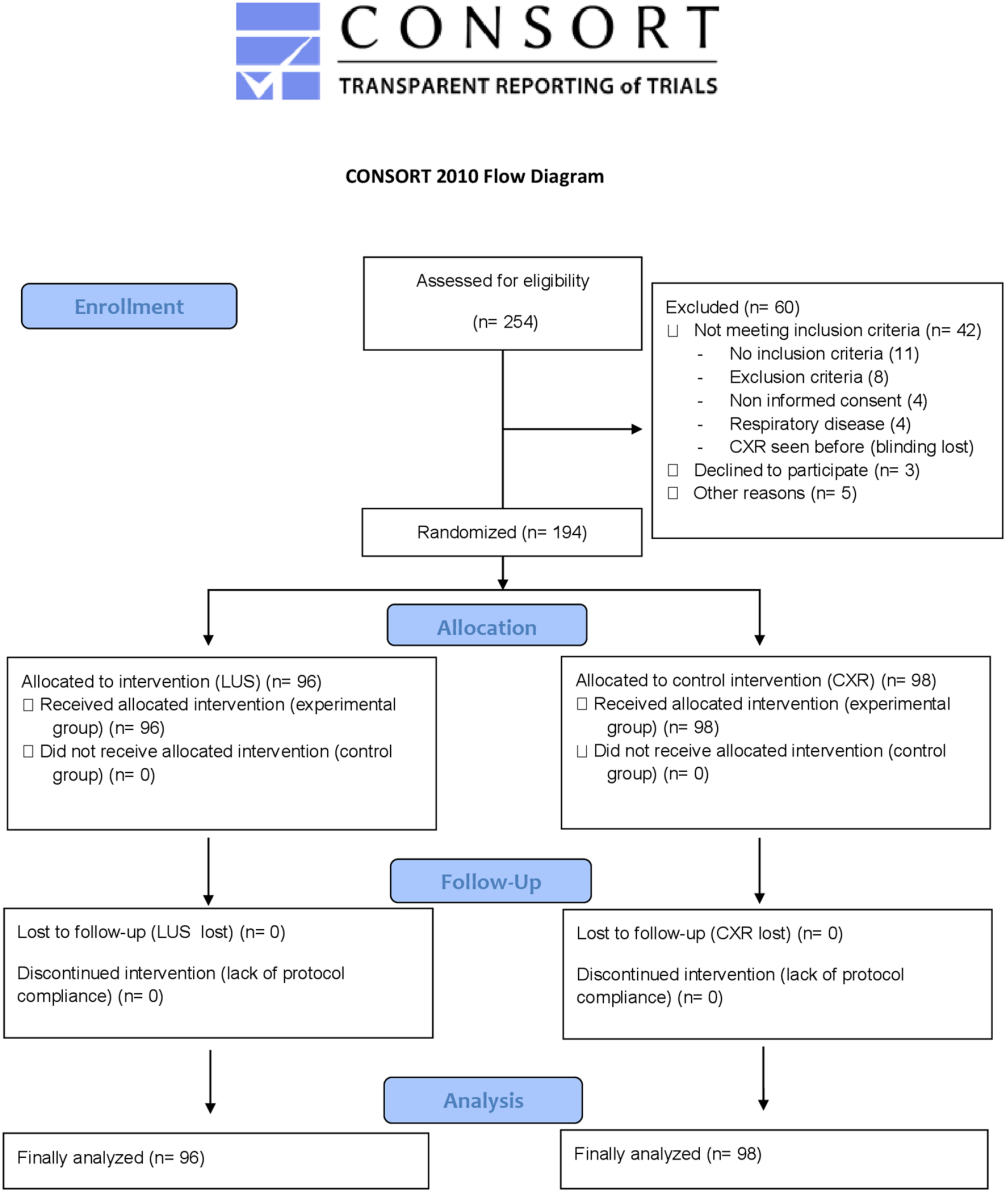
CONSORT flow diagram

Patients who met the inclusion criteria were randomly assigned to two groups (Fig. 1):

- Experimental group (EG): patients in this group underwent LUS as the first lung image test at the time of recruitment.
- Control group (CG): patients in this group underwent CXR as a first lung image test at recruitment.

The decision to prescribe antibiotic therapy was based on a combination of the imaging test results and the PCT value. The antibiotic therapy recommendations for each group were as follows:

1. If the image test (LUS or CXR) was not suggestive of BP and the PCT value was < 1 ng/mL, no antibiotic was prescribed.
2. If the image test (LUS or CXR) was suggestive of BP, regardless of the PCT value, antibiotic therapy was recommended.
3. If the image test (LUS or CXR) was not suggestive of BP but the PCT value was > 1 ng/mL, an empirical antibiotic was recommended to ensure the treatment of other infectious etiologies.

LUS was performed every day starting from recruitment, and the results were recorded and stored at three specific time points: within 12 h of suspicion of BP (basal visit), between the 2nd and 3rd day (first visit), and between the 4th and 5th day (second visit). Additionally, during the hospitalization period, LUS and CXR were performed based on the patient's clinical progress.

The pediatric researcher who conducted and interpreted the LUS, as well as the radiologist who interpreted the CXR, were kept unaware of each other’s findings and were also blinded to the final diagnosis assessment.

The final diagnosis of BP was determined by an expert using the BTS guidelines [3] for CAP taking in to consideration clinical, laboratory, microbiological, and radiological findings). The CDC criteria were followed for NP final diagnosis [38]. To analyze the agreement and accuracy of the tests, all patients underwent both CXR or LUS at the time of recruitment. Results can be found in previously published articles in the literature [35, 39].

### Economic evaluation

An economic evaluation was conducted. All resources involved in the use of the algorithm were included as direct costs: price per each imaging test, antibiotic saving costs, and CXR saving costs. No indirect costs were evaluated.

### Outcomes

The main outcome was to assess whether the algorithm, combining LUS and PCT, was useful for pneumonia management considering:

- The indication and duration of the empirical antibiotic treatment between groups:
  - The antibiotic indication, considered appropriate when patients completed a minimum of 5-day treatment [54], as the bacterial infection was confirmed.
  - The antibiotic prescription rate, evaluated through the concordance analysis between the final diagnosis (BP, VP, no pneumonia) and the antibiotic indication.
  - The antibiotic-free days, calculated as the difference between the global stay minus the total of the antibiotic days.
- To quantify the radiation dose avoided between groups.
- To quantify the time needed and complications for each imaging test.
- To determine if there was an economic impact regarding the image test.

The secondary outcome was to analyze if the implementation of the algorithm had an impact on avoided radiation on all other PICU patients. Two-time points were analyzed: 2015 and 2020, before and after the algorithm implementation. All patients admitted to the PICU due to a medical condition were considered. Surgical patients were excluded. The total CXR that were done before and after the protocol application, were compared. The CXR/patient ratio and the radiation/patient ratio were calculated and compared.

### Sample size and statistical analysis.

Using the statistical program Ene 2.0®, the sample size was estimated as sufficient to guarantee power of 80% with a level of significance of 5% to detect an effect size of 20% using a bilateral X^2^ test for two independent samples [33]. It resulted in a total of 182 units, 91 units per each group, EG and CG [33].

Using the "Random'' function of the MS-Excel XP® program (Microsoft ® Excel ® 2019), a binary series of random numbers was generated, according to the procedure described by Friedman, which creates the sequence by means of balanced block sampling to ensure a similar number of patients in each group. The series of numbers were in the possession of the PICU’s head researcher. Depending on that number, each patient was assigned to one group or the other.

The proportions of categorical variables were compared using the X^2^ test and the means of continuous variables were assessed with the Student’s T-test. A *p*-value of < 0.05 was considered statistically significant. The time of realization of each technique was described by medians and quartiles. Medians comparison was assessed using Wilcoxon’s rank test. Qualitative variables were described by counts and percentages.

Concordance between antibiotic prescription and the final diagnosis was evaluated using the Kappa index. It assessed the equality of concordance level by comparing the Kappa indices of each group using Wald’s *Z*-test. The association between the antibiotic prescription and the randomization group was tested by applying the Chi-square test or Fisher’s exact test if applicability conditions were not met. The antibiotic treatment time was described by medians and quartiles. The randomization group’s medians of time of antibiotic treatment were compared by the Mann–Whitney’s test. A *p*-value lower than 5% was considered significant.

## Results

A total of 254 children were assessed for study eligibility. 194 children were finally enrolled, and randomized into the two groups: 96 patients in the EG and 98 in the CG. The CONSORT flow diagram is shown in Fig. 1. Of the 194 patients, there were 113 (58.2%) females with a median age of 134 days (IQR 39–554), and a median weight of 6.3 kg (IQR 4.3–11.0). Demographic, clinical characteristics, and microbiological results were similar in both groups. Data are shown in Additional file 1 and Additional file 2, respectively.

### Primary outcome: management of BP suspicion

**Fig. 2:**
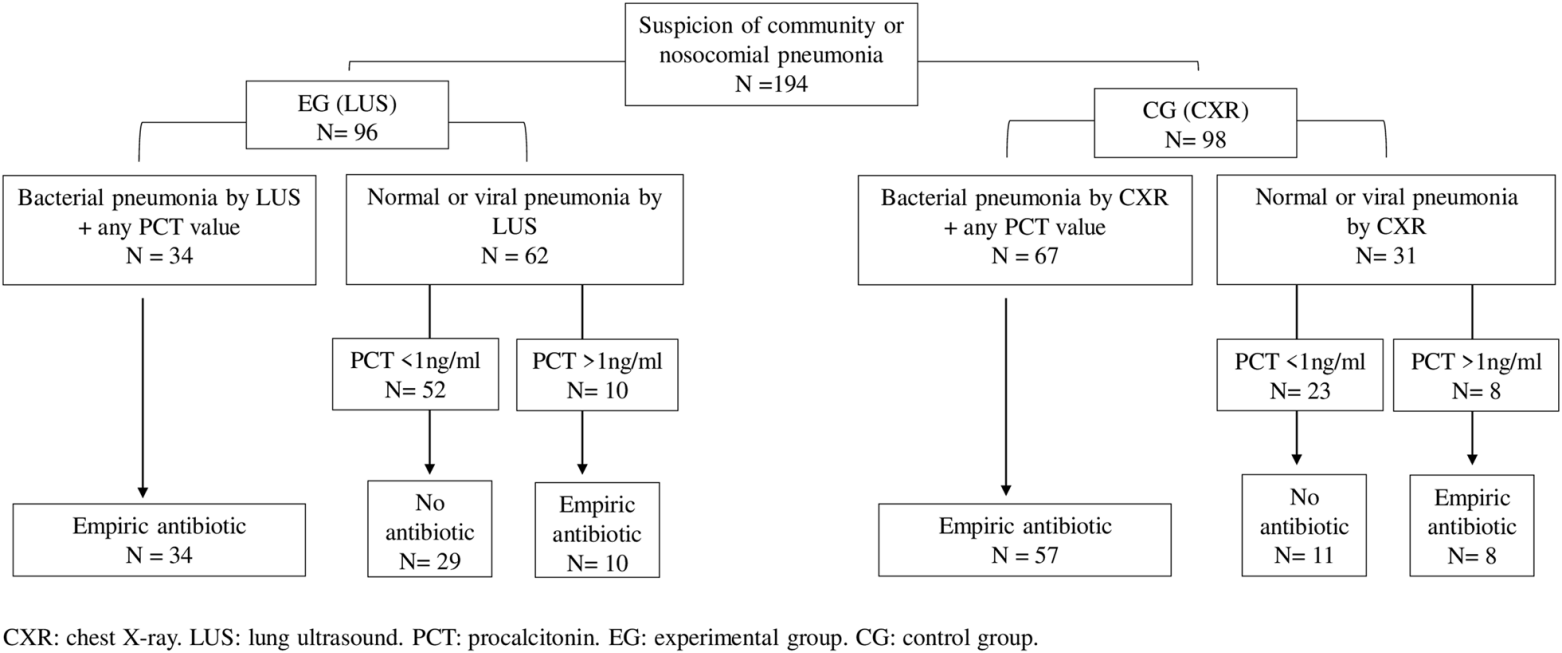
Results of the algorithm protocol implementation

Antibiotic indication and duration per each group at admission:

1. In 75/194 (38.7%) patients the image test was not suggestive of BP with PCT < 1 ng/ml; from them, 29/52 (55.8%) in the EG and 11/23 (47.8%) in the CG did not receive antibiotics.
2. In 101 patients, the image test was suggestive of BP; from them, 34/34 (100%) in the EG and 57/67 (85%) in the CG received antibiotics. Statistically significant differences between groups were observed when PCT resulted < 1 ng/ml, *p* = 0.01.
3. In 18 patients the image test was not suggestive of BP but PCT resulted in > 1 ng/ml. All of them received antibiotics. Details are shown in Fig. 2 and Table 1.

**Table 1:** Analysis of the antibiotic indication

Image findings PCT value	Basal visit			First visit			Second visit		
	*n*	A (%)	*p*	*n*	A (%)	*p*	*n*	A (%)	*p*
LUS N + PCT < 1	52	23 (44.2)	*0.59*	52	17 (32.7)	*0.44*	52	17 (32.7)	*0.44*
CXR N + PCT < 1	23	12 (52.2)		23	9 (39.1)		23	9 (39.1)	
LUS a + PCT > 1	17	17 (100)	*0.28*	17	16 (94.1)	*0.21*	17	16 (94.1)	*0.21*
CXR a + PCT > 1	25	25 (100)		25	25 (100)		25	25 (100)	
LUS a + PCT < 1	17	17 (100)	*0.07*	17	17(100)	*0.01*	17	17 (100)	*0.01*
CXR a + PCT < 1	42	32 (76.9)		42	26 (61.9)		42	26 (61.9)	
LUS N + PCT > 1	10	10 (100)	*0.81*	10	10 (100)	*0.45*	10	10 (100)	*0.45*
CXR N + PCT > 1	8	8 (100)		8	7 (75)		8	7 (75)	

The first column of each visit shows the number of patients with the combination of the imaging test and PCT results. The second column shows the number of patients who received antibiotic and the percentage it represents

LUS: lung ultrasound, CXR: chest X-ray, PCT: procalcitonin, n: number, A: antibiotic, N: normal, a: altered (bacterial pneumonia pattern by image test)

The median length of antibiotic therapy was of 7 days (IQR 7–7) in the EG *vs* 7 days (IQR 5.75–7) in the CG, *p* = 0.616. Antibiotic-free days resulted in a median of 9 days (IQR 4–15.5) for the LUS group *vs* 9 days (IQR 4–18.5) for the CXR group, *p* = 0.438.

The analysis of the antibiotic treatment for each group and visit is shown in Tables 2, 3, 4 and 5.

**Table 2:** Antibiotic indication and length

Visit	Total (*N* = 194)	EG (*N* = 96)	CG (*N* = 98)	*p* value
ATB basal visit, *n* (%)	144 (74.2)	67 (69.8)	77 (78.6)	0.217
ATB first visit, *n* (%)	127 (65.9)	60 (62.5)	67 (68.4)	0.479
ATB second visit, *n* (%)	127 (65.9)	60 (62.5)	67 (68.4)	0.479
Patients with final diagnosis of BP, *n* (%)	97 (50)	46 (47.9)	51 (52)	0.339

Patients who received antibiotic treatment for each group and visit

**Table 3:** Analysis of antibiotic duration in patients who received antibiotic

Antibiotic duration	Total (*N* = 144)	EG (*N* = 67)	CG (*N* = 77)	*p* value
< 3 d, *n* (%)	17 (11.8)	7 (10.5)	10 (12.9)	0.556
4–5 d, *n* (%)	19 (13.3)	9 (13.4)	10 (13.2)	
6–7 d, *n* (%)	81 (56.6)	42 (62.7)	39 (51.3)	
> 7 d, *n* (%)	28 19.6)	10 (14.9)	18 (23.7)	

Comparison between both experimental and control group

**Table 4:** Sub-analysis excluding those patients who received antibiotic with VP or normal pattern in the image test and PCT < 1 ng/ml, for each group and visit

Visit	Total (*N* = 109)	EG (*N* = 44)	CG (*N* = 75)	*p* value
ATB basal visit, *n* (%)	109 (100)	44 (100)	65 (86.7)	0.013
ATB first visit, *n* (%)	100 (91.7)	43 (97.7)	57 (76)	0.004
ATB second visit, *n* (%)	100 (91.7)	43 (97.7)	57 (76)	0.004
Patients with final diagnosis of BP, *n* (%)	88 (80.7)	41 (93.2)	47 (62.7)	0.323

**Table 5:** Sub-analysis of antibiotic duration, excluding those patients who received antibiotic with VP or normal pattern in the image test and PCT < 1 ng/ml

Antibiotic duration	Total (*N* = 109)	EG (*N* = 44)	CG (*N* = 75)	*p* value
< 3 d, *n* (%)	9 (0.1)	1 (2.3)	8 (12.3)	0.126
4–5 d, *n* (%)	8 (0.1)	2 (4.6)	6 (9.2)	
6–7 d, *n* (%)	69 (63)	33 (75)	36 (55.4)	
> 7 d, *n* (%)	23 (21)	8 (18.2)	15 (23.1)	

#### Antibiotic prescription analysis

Of the 194 patients with suspected BP, 144 (74.2%) received empirical antibiotic treatment at the basal visit, 67/96 (69.8%) patients in the EG, and 77/98 (78.6%) in the CG (*p* = 0.217).

BP was finally diagnosed in 97 (50%) patients, 46/96 (47.9%) in the LUS group, and 51/98 (52%) in the CXR group (*p* = 0.816). Of them, 45/46 (97.8%) in the LUS group, and 50/51 (98%) in the CXR group, received antibiotic treatment (*p* = 0.339).

A sub-analysis, excluding patients with normal viral pattern in the image test and PCT < 1 ng/ml), showed that 44/44 patients in the EG and 65/75 in the CG (p0.01), received antibiotics. Details are shown in Table 4.

The concordance analysis between the final diagnosis of BP and the antibiotic treatment prescription resulted in *K* 0.67 (IC 95% 0.529–0.811) for the LUS group, and in *K* 0.627 (IC 95% 0.48–0.774) for the CXR group, *p* = 0.339.

#### The pros and cons of LUS vs CXR

##### Avoided radiation

Each anteroposterior CXR irradiates 0.02mSv [10]. All patients underwent CXR on the day of admission (194 CXR). During the follow-up, 30 CXR (12 in the EG and 18 in the CG) were done between 24 and 48 h and, 17 CXR (9 in the EG and 8 in the CG) were done after the fifth day. So, 164 and 177 CXR were eluded during patient follow-up, a total of 341 CXR. Hence, a total of 6.82 mSv radiation was avoided, meaning 0.035 mSv radiation eluded per patient.

##### Time and complication for each imaging test

The median time spent for the LUS procedure resulted in 7 min (IQR 6–8) *vs* a median time of 31 min (IQR 19–65.8) for CXR, with statistically significant differences (*p* < 0.001). The CXR time was calculated from the CXR solicitude until it was able to be seen on the computer.

Regarding LUS, complications were defined in 19 (9.9%) patients: 11 (5.67%) needed sedation increase, 5 (2.58%) needed increase of oxygen administration, in 2 (1.0%) occurred desaturation and in 1 (0.5%) bradycardia. About CXR, there were observed complications in 44 (22%) patients: 21 (10.8%) needed sedation increase, 16 (8.2%) needed increased oxygen administration, 5 (2.6%) presented bradycardia and 1 (0.5%) had accidental extubating. Complications between imaging tests had statistically significant differences (*p* < 0.01).

##### Costs analysis

By the rate of the Catalonian Government decree (CVE-DOGC-B-20155037-2020) [55], the price per each LUS was set at 24.8$ and the price per each CXR, at 15.5$. As LUS was done at the patient’s bedside by the same physician in charge of the patient, it had no further human costs. By the rate of Hospital costs, antibiotic costs per day were calculated. The total antibiotic cost in PICU, during one year, was 30805,8$. PICU days of stay were 5894 days. Antibiotic costs resulted in 5.17$ per day-present. Table 6 summarizes all costs.

**Table 6:** Cost analysis regarding image test and antibiotic prescription

	Total *N* = 194	EG *N* = 96	CG *N* = 98	*p*-value
CXR				
*n*, %	247 (100)	116 (47)	131 (53)	
mean (DS)	–	1.21 (0.43)	1.34 (0.63)	0.076
Total CXR costs (€)	3705	1740	1965	
LUS				
*n*, %	507 (100)	250 (49)	257 (51)	0.819
Mean (DS)	–	2.60 (0.66)	2.62 (0.58)	
Total LUS costs (€)	12168	6000	6168	
(A) Total image costs (€)	15873	7740	8133	

	Total *N* = 194	EG *N* = 96	CG *N* = 98	*p*-value
Antibiotic				
*n*, %	144 (74.2)	67 (69.8)	77 (78.6)	0.217
Duration, median (IQR)	7 (6–7)	7 (7–7)	7 (5–7)	
Total days of antibiotic	1008	469	539	
(B) Total antibiotic costs (€)	5040	2325	2695	
(A + B) Total costs (€)	20913	10065	10828	

### Secondary outcome: analysis before and after algorithm implementation

The total number of CXRs done before and after the protocol application was compared. 741 CXRs were done in 2015 and 645, in 2020. The total number of medical patients admitted to the PICU was 642 and 661, respectively, an increase of 2.8% of medical patients. A reduction of 12.9% of CXR was observed, meaning a total of 0.96 mSv was avoided. The CXR/patient ratio was 0.27 CXR/patient during 2015 and 0.06 CXR/patient during 2020. The radiation/patient ratio was 0.41 for 2015 and 0.45 for 2020. A reduction of 77% CXR/patient was observed.

The reduction of 12.9% of CXR before and after the algorithm application is supposed to save a total of 2579$.

## Discussion

The algorithm presented has already demonstrated that it is possible to improve the accuracy and concordance for BP diagnosis when combining LUS and PCT [35]. Besides, it might improve the BP management, regarding antibiotic indication, avoiding-radiation without increasing costs.

There are scarce data regarding the combination of LUS and PCT for pneumonia diagnosis in children. Some papers have analyzed LUS and PCT regarding their accuracy for bacterial pneumonia diagnosis in adult population [56, 57]. Our research group has also analyzed it in children, resulting in better accuracy when combining LUS with PCT than CXR with PCT [35]. However, no previous studies have analyzed the impact of the algorithm implementation on antibiotics indication, avoided radiation, or costs. L. Lhopitallier et al. [34] pointed out that a minority of adult patients presenting with lower respiratory tract infection (LRTI) have CAP and require antibiotic therapy. Identifying these patients is challenging because of overlapping symptomatology and the low diagnostic performance of CXR. They suggested that PCT could be safely used to indicate antibiotic prescription in patients with LRTI. Moreover, they also considered LUS effective in detecting lung consolidation in pneumonia, which might compensate the lack of PCT specificity [34]. This situation may also occur in the pediatric population. Therefore, combining PCT and LUS could be a reliable point-of-care strategy to reduce antibiotic prescription in LRTIs without impacting patients' safety. Their results [58] regarding the analysis of LUS and PCT to decide on antibiotic prescription in adult patients with CAP showed that compared with the usual care, PCT led to a 26% absolute reduction in the probability of 28-day antibiotic prescription without affecting patients’ safety [58]. However, the potential added value regarding the addition of the LUS result could not be perfectly evaluated as they only performed lung ultrasonography in patients with elevated PCT [58].

Compared to other studies where patients from the pediatric ward and emergency department are included, our study included only those patients admitted to the PICU. This bias may lead to different results as our patients require mechanical respiratory support and therefore were much sicker. Moreover, the median age of the patients was very young compared to other studies, which may be because younger patients with a respiratory infection may develop severe respiratory insufficiency requiring more respiratory support, and consequently, PICU admission.

It is well known that frequent inappropriate antibiotic prescription occurs in pediatric ICU population [59], due to the existence of other non-bacterial acute respiratory infections/diseases, for example, acute bronchiolitis, that may lead to uncertain diagnosis because of the non-specific clinical presentation of patients with concomitant BP [60]. Based on our results, we speculate that the new algorithm could be safe to ensure no antibiotic prescription when both image test and PCT levels are normal. Another consideration could be that, in those patients with clinical signs and symptoms suggestive of bacterial infection at admission, antibiotics could be initiated. However, if the algorithm resulted in neither image test nor PCT values suggestive of infection, the algorithm could lead to antibiotic discontinuation within 24–48 h in those patients with favorable evolution.

On the other hand, when LUS was suggestive of BP, regardless of PCT value, antibiotic therapy was indicated in all patients (34/34, 100%). However, when CXR was suggestive of BP, a proportion of 15% (10/57) of patients did not receive empirical antibiotic therapy. All those patients with suggestive BP pattern in CXR who did not receive antibiotic treatment had a PCT value < 1 ng/ml. None of them needed to initiate antibiotics in the following days. Due to hesitation about the CXR consolidation, no empirical antibiotic was initiated, probably because of the low CXR specificity regarding BP diagnosis [61, 62]. Observing the sub-analysis results presented in Table 4, it could be inferred that if physicians had rigorously followed the algorithm, the CXR would have led to an antibiotic overtreatment with statically significant differences.

Despite several attempts, the etiologic diagnosis of pediatric CAP and the estimation of the potential outcome remain unsolved problems in most cases. Some data are available on the usefulness of combined clinical and laboratory-based algorithms for screening children with respiratory infections who need antibiotics and likewise for the selection of an appropriate antibiotic [63]. Among traditional biomarkers, procalcitonin (PCT) appears to be the most effective for both selecting bacterial cases and evaluating the severity, being widely studied and used in pediatrics. Principi N et al. reported that only PCT concentrations ≥ 1 ng/mL, were a reliable marker of bacterial CAP [64]. Another Italian study [65], which included 319 children hospitalized for uncomplicated CAP, randomized patients to treatment guided by PCT and to standard care. The PCT-guided group received fewer antibiotic prescriptions (85.8% vs. 100%), and the group was on average exposed to antibiotics for a shorter time (5.4 vs. 11.0 days). The results of our study showed that when the image test (LUS or CXR) was not suggestive of BP, but the PCT value was > 1 ng/mL, an empirical antibiotic was indicated in 100% of the patients in both groups, to ensure the treatment of other infectious etiologies. Of these patients, 13 were finally diagnosed with clinical-analytical sepsis without microorganism isolation, 4 had urinary tract infections, and 1 presented with bronchoaspiration.

In both groups, notwithstanding the PCT value < 1 ng/ml in the branch of normal/viral pattern, up to 44.2% and 52.1% of the patients in the LUS and CXR groups, respectively, received empirical antibiotics. It might be due to the existence of other non-bacterial acute respiratory infections/diseases, with non-specific overlapped clinical presentation. This could be the reason why no antibiotic reduction was seen comparing both groups in general. Even though, among those patients, 84% in the EG and 73% in the CG, were not finally diagnosed with bacterial pneumonia or had other bacterial infections. So, we could speculate that the new algorithm could be safe to ensure no antibiotic prescription when both image test and PCT levels are normal.

Regarding radiation, the International Commission for Radiation Protection (ICRP) system of radiation protection is based on three fundamental principles: justification, optimization, and dose limitation [66]. So that, the introduction of a radiation source should result in sufficient individual or societal benefit to offset the detriment it causes [66]. The results of our study showed a reduction of individual and global radiation, not only for patients with suspected pneumonia but indirectly for all PICU patients. A randomized control trial [67] comparing LUS with CXR in children with suspected pneumonia, also showed up to 60.6% reduction in CXR use, with no cases of missed pneumonia among all study participants, nor differences in adverse events. Jones BP et al [67], concluded that it might be feasible and safe to substitute LUS for CXR when evaluating children suspected of having pneumonia with no missed cases of pneumonia or increase in rates of adverse events. As radiography uses ionizing radiation [68], it must be used judiciously. But not having follow-up imaging may reduce case ascertainment in research studies [69, 70]. The LUS may be the imaging modality of choice for the diagnosis of pneumonia in all healthcare settings [69, 70].

Concerning costs, Zhang et al. analyzed the costs of management per episode of pneumonia and stratified them by income-country category. They defined that in high-income countries, the average costs of facility-based case management for young children with pneumonia admitted in tertiary hospitals were 7073.2 US$ (95% CI 4028.6–11311.0) [71]. The cost of antibiotic treatment for all children with pneumonia in 66 countries in the countdown to 2015, for maternal, new-born, and child survival was estimated at around US$ 109 million per year. The price included the antibiotics and diagnostic tests for pneumonia management [72]. Our study analyzed only direct costs, regarding image tests and antibiotic treatment, resulting in a total of 21608,7$ during the study period.

The implementation of radiography equipment is expensive [68]. Image quality and technician training also affect interpretation. Training and maintaining study staff with close adherence to a standardized protocol and frequent re-standardization and quality control to mitigate high inter-reader variability is high-priced [73]. LUS might be more time and resource efficient by not increasing patient costs and by decreasing the time spend doing the technique and analyzing the results [74]*.*

POCUS (point-of-care ultrasound) [39, 75, 76] involves a focused assessment and information that can be integrated with clinical and laboratory data, making timely and accurate decisions possible. Following POCUS guidelines may help in standardizing clinical practices in acute care settings, helping evaluate pneumonia and pleural effusions in infants and children [76]. Radioprotection, manageability, and a more targeted use of image tests, when performed by the physician in charge of the patient, are some of the most important advantages LUS has over CXR [77, 78]. Even though, the presence of lobar consolidation [80] might be indicative of bacterial disease, CXR does not provide information about the causes of pneumonia [79]. LUS features might be able to predict pneumonia etiology in children [81].

During the last 10 years, no new antibiotics have been launched for BP in children. The antibiotic use should be limited and better targeted [63]. This goal could be reached by developing evidence-based algorithms for the diagnosis and treatment of BP and other respiratory infections in children [27, 63]. Effective launching, informing and monitoring strategies are needed. Optimally, algorithms containing clinical findings, clinical measurements, results of point-of-care biomarkers, and image tests, such as microbe detection tests [82], in the future, may be based on large local data modified online by machine learning [83].

## Limitations

Our study has several limitations. Firstly, it was conducted in a single center, which may limit the generalizability of the findings to other settings. Secondly, the accuracy of microbiological diagnoses of pneumonia is not always 100% reliable. This is because obtaining samples through invasive procedures like TA/BAL is not feasible for non-mechanically ventilated children. Additionally, alternative diagnostic tests such as PCR may have lower Sn [82]. Thirdly, the estimated antibiotic costs were based on data from the hospital’s dispensing pharmacy, which may not perfectly reflect the actual antibiotic administrations due to potential variations.

Finally, while CT scan may be considered ideal for diagnosing pneumonia, it is not practical or ethical for safety concerns and radiation exposure, especially considering the potentially long life expectancy of these patients. CT scans are typically reserved for special cases involving poor clinical evolution, complicated pneumonia with parapneumonic effusions, necrotizing pneumonia, or lung abscesses. Therefore, in this study, the final diagnosis was made by the expert based on the BTS guidelines and CDC definition, without relying on CT scans.

## Conclusions

The use of LUS and PCT, showed no risk of mistreating BP. The algorithm ensued in similar results regarding antibiotic treatment indication but avoiding radiation exposure and without increasing costs. Therefore, the algorithm could be a reliable tool for pneumonia management in critically ill children.

### Supplementary Information

**Additional file 1.** Demographic and clinical variables.

**Additional file 2.** Microbiological samples and results.

## Data Availability

Requests for data should be made to the corresponding author. Each request requires a research proposal including a clear research question and proposed analysis plan. Requests will be considered on an individual basis and are subject to review and approval by the PROLUSP management committee and relevant human research ethics committees.

## Abbreviations

BAL: Bronchoalveolar lavage
BP: Bacterial pneumonia
BTS guidelines: British Thoracic Society guidelines
CAP: Community-acquired pneumonia
CDC: Centers for Disease Control and Prevention
CG: Control group
C-RP: C-reactive protein
CXR: Chest X-ray
EG: Experimental group
LUS: Lung ultrasound
NPA: Nasopharyngeal aspirate
NP: Nosocomial pneumonia
PCR: Polymerase chain reaction
PCT: Procalcitonin
PICU: Pediatric intensive care unit
POCUS: Point-of-care ultrasound
TA: Tracheal aspirate
VP: Viral pneumonia

## Code availability

Requests for code should be made to the corresponding author and will be considered on an individual basis by the study management committee.
