# Supplementary material for: Lung ultrasound and procalcitonin, improving antibiotic management and avoiding radiation exposure in pediatric critical patients with bacterial pneumonia: a randomized clinical trial
Source: Eur J Med Res. 2024 Apr 6;29:222. doi: 10.1186/s40001-024-01712-y (PMC10998368; doi:10.1186/s40001-024-01712-y)
Supplement: Supplementary file 1 — Additional file 1. Demographic and clinical variables. [file 40001_2024_1712_MOESM1_ESM.doc]

Additional file 1. Demographic and clinical variables.

|  | **TOTAL**  N = 194 | **EG (LUS)**  N = 96 | **CG (CXR)**  N = 98 | **p- value** |
| --- | --- | --- | --- | --- |
| Gender (female), n (%) | 113 (58.2) | 52 (54.2) | 61 (62.2) | 0.320 |
| Age (days), median (IQR) | 134 (39-554) | 113 (42.2-486) | 156 (34.5-578) | 0.949 |
| Weight (kg), median (IQR) | 6.30 (4.3-11.0) | 6.30 (4.4-10.0) | 6.40 (4.35-12.0) | 0.679 |
| Comorbidities, n (%)  None  Respiratory  Cardiovascular  Infectious diseases  Neurological  Hematology-oncology  Other | 116 (59.8)  31 (16.0)  15 (7.7)  2 (1.0)  7 (3.6)  2 (1.0)  21 (10.8) | 58 (60.4)  15 (15.6)  6 (6.3)  1 (1.0)  4 (4.2)  1 (1.04)  11 (11.5) | 58 (59.2)  16 (16.3)  9 (9.%)  1 (1.0)  3 (3.1)  1 (1.0)  10 (10.2) | 0.985 |
| Reason for admission, n (%)  Infection^#^  Trauma  CV surgery/ Heart failure  Surgery (Abd, Trauma, NS)  Other | 149 (76.8)  7 (3.61)  9 (4.64)  4 (2.06)  25 (12.9) | 77 (80.2)  3 (3.12)  5 (5.21)  0 (0.00)  11 (11.5) | 72 (73.5)  4 (4.08)  4 (4.08)  4 (4.08)  14 (14.3) | 0.342 |
| Respiratory failure, n (%) | 159 (82.0) | 79 (82.3) | 80 (81.6) | 1.000 |
| Severity upon admission,  PRISM, median (IQR) | 2 (0-5) | 2 (0-4) | 3 (0-5) | 0.123 |
| Length of stay  PICU (days), median (IQR)  Hospitalization (days), median (IQR) | 7.0 (4.0 -14.7)  26.0 (17.5-43.5) | 7.0 (4.0-14.0)  25.0 (12.2-40.0) | 8.0 (5.0-15.0)  29.0 (18.0-43.0) | 0.350  0.117 |
| Respiratory support  HFNC, n (%)  NIV, n (%)  CMV, n (%)  NO, n (%) | 103 (53.1  166 (85.6%)  85 (43.8)  9 (4.6) | 54 (56.2)  88 (91.7)  36 (37.5)  3 (3.1) | 49 (50.0)  78 (79.6)  49 (50.0)  6 (6.1) | 0.466  0.029  0.107  0.498 |
| Inotropic support, n (%) | 23 (11.9) | 9 (9.38) | 14 (14.3) | 0.403 |
| ECMO, n (%) | 3 (1.6) | 2 (2.08) | 1 (1.02) | 0.619 |
| Death, n (%) | 1 (0.52) | 1 (1.04) | 0 (0.00) | 0.495 |
| Bacterial pneumonia, n (%) | 97 (50) | 46 (47.9) | 51 (52.0) | 0.816 |
| Leukocytes, median (IQR)  CRP mg/L, median (IQR)  PCT ng/dl, median (IQR) | 10800 (7500-15575)  43.1 (20.0-96.1)  0.60 (0.18-2.26) | 11000 (8300-16050)  37.8 (13.0-87.9)  0.54 (0.14-3.32) | 10100 (6800-15050)  50.0 (24.1-105)  0.65 (0.19-1.72) | 0.159  0.110  0.637 |
| Antibiotic, n (%) | 144 (73.7) | 67 (96.8) | 77 (78.5) | 0.667 |

#Infection: meningitis, pneumonia, sepsis, bronchiolitis. IQR: interquartile range. CV: cardiovascular. Abd: abdominal surgery. NS: neurosurgery. PICU: pediatric intensive care unit. HFNC: high flow nasal cannula. NIV: non-invasive ventilation. CMV: conventional mechanical ventilation. NO: nitric oxide. ECMO: extracorporeal membrane oxygenation. CRP: C-reactive protein. PCT: procalcitonin, BP: bacterial pneumonia; VP: viral pneumonia. Complete Antibiotic: patients who completed antibiotic treatment with a final diagnosis of bacterial infection.
