# Supplementary material for: Lung ultrasound and procalcitonin, improving antibiotic management and avoiding radiation exposure in pediatric critical patients with bacterial pneumonia: a randomized clinical trial
Source: Eur J Med Res. 2024 Apr 6;29:222. doi: 10.1186/s40001-024-01712-y (PMC10998368; doi:10.1186/s40001-024-01712-y)
Supplement: Supplementary file 2 — Additional file 2. Microbiological samples and results. [file 40001_2024_1712_MOESM2_ESM.docx]

Additional file 2. Microbiological samples and results.

|  | **TOTAL**  N = 194 | **EG (LUS)**  N = 96 | **CG (CXR)**  N = 98 | p-value |
| --- | --- | --- | --- | --- |
| **Viral multiplex PCR, n (%)** | 169 (87.1) | 83 (86.4) | 86 (87.8) | 0.913 |
| **Viral result, n (%)**  RSV  Influenza A  Influenza B  Influenza A/H1N1  Metapneumovirus  Rinovirus/Enterovirus  Parainfluenza  Mycoplasma  Adenovirus  Coronavirus | 101 (50.2)  6 (2.99)  1 (0.5)  1 (0.5)  6 (2.99)  71 (35.3)  3 (1.49)  0 (0)  6 (2.99)  6 (2.99) | 48 (49)  3 (3.06)  0 (0)  0 (0)  5 (5.1)  35 (35.7)  1(1.02)  0 (0)  4 (4.08)  2 (2.04) | 53 (51.5%)  3 (2.91%)  1 (0.97%)  1 (0.97%)  1 (0.97%)  36 (34.9)  2 (1.04)  0 (0)  2 (1.94)  4 (3.88) | 0.717 |
| **Viral coinfection, n (%)** | 47 (24.2) | 23 (24) | 24 (24.5) | 1.000 |
| **NFA, n (%)** | 105 (54.1) | 54 (56.2) | 51 (52) | 0.657 |
| NFA result, n (%)  Negative  H. influenzae  M. catarrhalis  S. pneumoniae  S. aureus  S pyogenes  E. coli  K. pneumoniae  Enterobacter  Serratia  N. meningitidis  MRSA  H. parainfluenzae | 33 (17)  44 (22.7)  20 (10.3)  14 (7.2)  7 (3.6)  0 (0)  6 (3.1)  2 (1)  2 (1)  2 (1)  1 (0.5)  2 (1)  1 (0.5) | 21 (63.6)  20 (45.4)  9 (45)  7 (50)  5 (71.4)  0 (0)  3 (50)  2 (100)  0 (0)  0 (0)  0 (0)  0 (0)  1 (50) | 12 (36.4)  24 (54.6)  11 (65)  7 (50)  2 (28.6)  0 (0)  3 (50)  0 (0)  2 (100)  2 (100)  1 (100)  2 (100)  0 (0) | 0.768 |
| **TA/BAL, n (%)** | 65 (33.5) | 26 (27.1) | 39 (39.8) | 0.085 |
| **TA/BAL result, n (%)**  Negative  H. influenzae  M. catarrhalis  S. pneumoniae  S. aureus  S. pyogenes  E. coli  K.pneumoniae  Enterobacter  Serratia  N meningitidis  MRSA  Candida  H parainfluenza  K. oxitoca  S. viridans  Pseudomonas  Stenotrophomonas  A.Bereziniae | 17 (8.8)  14 (7.2)  7 (3.6)  10 (5.2)  8 (4.1)  1 (0.5)  2 (1)  6 (3.1)  1 (0.5)  6 (3.1)  0 (0)  1 (0.5)  0 (0)  0 (0)  4 (2.1)  1 (0.5)  8 (4.1)  2 (1)  1 (0.5) | 4 (23.5)  9 (64.3)  4 (57.1)  3 (30)  4 (50)  1 (100)  1 (50)  2 (33.3)  1 (100)  2 (33.)  0 (0)  0 (0)  0 (0)  0 (0)  2 (50)  0 (0)  1 (12.5)  1 (50)  1 (100) | 13 (76.5)  5 (26.7)  3 (42.9)  7 (70)  4 (50)  0 (0)  1 (50)  4 (66.7)  0 (0)  4 (66.7)  0 (0)  1 (100)  0 (0)  0 (0)  2 (50)  1 (100)  7 (87.5)  1 (50)  0 (0) |  |
| **Blood culture, n (%)** | 173 (89.2) | 84 (87.5) | 89 (90.8) | 0.608 |
| **Blood culture result, n (%)**  Negative  S.pneumoniae  E.faecalis  K. oxytoca  K. pneumoniae  H.influenzae  Salmonela enterica  E. coli  MRSA  Serratia | 162 (90.6)  2 (1.16)  1 (0.58)  1 (0.58)  2 (1.16)  1 (0.58)  1 (0.58)  1 (0.58)  1 (0.58)  1 (0.58) | 79 (94.0)  0 (0)  1 (1.19)  0 (0)  2 (2.38)  1 (1.19)  0 (0)  1 (1.19)  0 (0)  0 (0) | 83 (93.3)  2 (2.25)  0 (0)  1 (1.2)  0 (0)  0 (0)  1 (1.2)  0 (0)  1 (1.2)  1 (1.2) | 0.167 |

LUS: lung ultrasonography; CXR: chest X-ray; RSV: respiratory syncytial virus; NFA: nasopharyngeal aspirate; TA/BAL: tracheal aspirate/bronchoalveolar lavage.
